# Natural Diet of Coral-Excavating Sponges Consists Mainly of Dissolved Organic Carbon (DOC)

**DOI:** 10.1371/journal.pone.0090152

**Published:** 2014-02-25

**Authors:** Benjamin Mueller, Jasper M. de Goeij, Mark J. A. Vermeij, Yannick Mulders, Esther van der Ent, Marta Ribes, Fleur C. van Duyl

**Affiliations:** 1 Department of Biological Oceanography, Royal Netherlands Institute for Sea Research, Den Hoorn, The Netherlands; 2 CARMABI, Willemstad, Curaçao; 3 Department of Earth Sciences, Utrecht University, Utrecht, The Netherlands; 4 Department of Aquatic Ecology and Ecotoxicology, University of Amsterdam, Amsterdam, The Netherlands; 5 Department of Aquatic Microbiology, University of Amsterdam, Amsterdam, The Netherlands; 6 Departament de Biologia Marina i Oceanografia, Institut de Ciències del Mar (ICM-CSIC), Barcelona, Spain; University of Genova, Italy

## Abstract

Coral-excavating sponges are the most important bioeroders on Caribbean reefs and increase in abundance throughout the region. This increase is commonly attributed to a concomitant increase in food availability due to eutrophication and pollution. We therefore investigated the uptake of organic matter by the two coral-excavating sponges *Siphonodictyon* sp. and *Cliona delitrix* and tested whether they are capable of consuming dissolved organic carbon (DOC) as part of their diet. A device for simultaneous sampling of water inhaled and exhaled by the sponges was used to directly measure the removal of DOC and bacteria *in situ*. During a single passage through their filtration system 14% and 13% respectively of the total organic carbon (TOC) in the inhaled water was removed by the sponges. 82% (*Siphonodictyon* sp.; mean±SD; 13±17 μmol L^−1^) and 76% (*C. delitrix*; 10±12 μmol L^−1^) of the carbon removed was taken up in form of DOC, whereas the remainder was taken up in the form of particulate organic carbon (POC; bacteria and phytoplankton) despite high bacteria retention efficiency (72±15% and 87±10%). *Siphonodictyon* sp. and *C. delitrix* removed DOC at a rate of 461±773 and 354±562 μmol C h^−1^ respectively. Bacteria removal was 1.8±0.9×10^10^ and 1.7±0.6×10^10^ cells h^−1^, which equals a carbon uptake of 46.0±21.2 and 42.5±14.0 μmol C h^−1^ respectively. Therefore, DOC represents 83 and 81% of the TOC taken up by *Siphonodictyon* sp. and *C. delitrix* per hour. These findings suggest that similar to various reef sponges coral-excavating sponges also mainly rely on DOC to meet their carbon demand. We hypothesize that excavating sponges may also benefit from an increasing production of more labile algal-derived DOC (as compared to coral-derived DOC) on reefs as a result of the ongoing coral-algal phase shift.

## Introduction

Coral-excavating sponges are usually the most abundant and destructive bioeroders on coral reefs and strong competitors for space [1], [2]. They account for 60 to >90% of total macroborer activity [3], [4] and can remove up to 30 kg CaCO_3_ m^−2^ year^−1^ [5], which is in the same range as coral reef calcification rates ([6] and references therein). Coral-excavating sponges thus influence the balance between reef accretion (calcification and cementation) and erosion (physical, chemical and bioerosion), whereby positive net accretion is crucial to maintain carbonate reef structures [7]. Coral reefs are increasingly subjected to anthropogenic disturbances that negatively impact the growth of calcifying organisms while favoring (bioeroding) suspension feeders [8]–[10]. This is of particular importance in the face of climate change, where rising seawater temperatures [11], [12] and ocean acidification [11], [13] are expected to further reduce calcification rates of these organisms. In turn, the same processes are expected to promote bioerosion or at least affect it to a lesser extent [14]–[17], thus further reducing the ability of reef communities to form and maintain three dimensional reef frameworks. Over the past three decades the abundance of excavating sponges has increased considerably, mostly tentatively linked to increased food availability (e.g., bacterioplankton and phytoplankton) in response to eutrophication and land-based pollution [8]–[10], [18]. Similar to non-excavating sponges, coral-excavating sponges are commonly assumed to be efficient suspension feeders [19], i.e. feeding on particulate food sources. Yet, apart from the contribution of photosynthetically-fixed carbon from symbiotic zooxanthellae to the nutrition of some coral-excavating sponges [16], [20], [21] little is known about their dietary composition and food uptake rates.

Traditionally, sponges were considered to be suspension feeders that efficiently remove bacterio-, phyto- [22]–[26] and even zooplankton [27] from water they actively pump through their filtration systems. However, already in 1974, Reiswig [28] hypothesized that sponges may also retain dissolved organic carbon (DOC), which was later confirmed for several sponges, ranging from tropical [29]–[31] to temperate sponge species [32]. These tropical coral reef sponges can take up >90% of the total organic carbon (TOC) as DOC, indicating that they foremost rely on DOC to meet their carbon demand [29], [30]. Since DOC also accounts for >90% of the TOC pool on coral reefs (e.g., [29]), the ability to utilize this food source may aid certain sponges to thrive under oligotrophic conditions, whereas most other heterotrophic reef organisms are unable to capitalize on this resource [31]. Therefore, the question arises if, and to what extent, coral-excavating sponges also rely on dissolved organic substances in their daily diet.

The dissolved organic matter (DOM) uptake of non-excavating sponges is estimated to be in the same order of magnitude as the gross primary production rates of entire coral reef ecosystems [31]. Moreover, they are at the base of a pathway that transfers the DOM into particulate detritus that is subsequently ingested by reef fauna. This sponge loop retains the energy and nutrients within the different reef communities and most likely affects the stable states of these communities. Coral-excavating sponges are not yet considered to participate in the sponge loop, of which the ability to feed on DOM is one of the prerequisites.

The often suggested importance of food availability to explain the current increase of coral-excavating sponges requires experimental proof, in particular to address (1) whether coral-excavating sponges, similar to non-excavating sponges, are capable of DOC uptake and, if confirmed, (2) to what extent it completes their total daily diet. To answer these questions we determined the uptake of DOC and bacteria by the common Caribbean coral-excavating sponges *Siphonodictyon* sp. (Berquist, 1965) and *Cliona delitrix* (Pang, 1973) *in situ* and estimated the respective contribution of DOC and POC (bacteria and phytoplankton) to their TOC uptake.

## Materials and Methods

### Ethics statement

Research on Curaçao was performed under the annual research permit (unnumbered) issued by the Curaçaoan Ministry of Health, Environment and Nature (GMN) to the CARMABI foundation. Research conducted on Bonaire was performed under research permit No. 2012004073 issued by the Bonaire National Marine Park (BNMP) authority.

### Study area and sampling procedure

**Figure 1:**
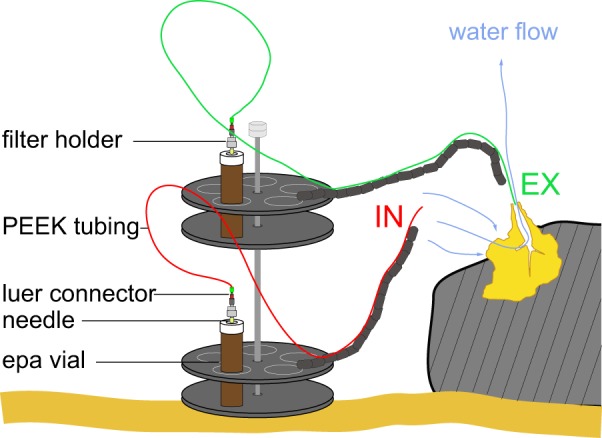
VacuSIP system for *in situ* sampling of DOC and bacteria. VacuSIP system consisting of two separate samplers (In and Ex) attached to a stand to simultaneously take water samples of the ambient water (IN) and the water exhaled by the sponge (EX). Blue arrows indicate water pumped through the sponge.

The study was conducted in May 2013 on the Southern Caribbean Islands of Curaçao and Bonaire (ESM table S1). *Siphonodictyon* sp. was sampled on the fore reef slope along the leeward coast of Curaçao at 19±1 m water depth (mean±SD) at stations Playa Jeremy (12° 33′ N, 69° 15′ W; *n* = 5) and Daaibooi (12° 21′ N, 69° 08′ W; n = 3). Both sites are characterized by narrow bays harboring a wide and sandy reef terrace (160–190 m) that leads to a fairly steep (>45°) fore reef slope off-shore [33]. Sampling of *Cliona delitrix* took place on the fore reef slope at 13±1 m water depth at station Playa Lechi (12° 16′ N, 68° 28′ W; n = 10) in front of Kralendijk, Bonaire. Here, the sandy reef terrace is narrow (approx. 65 m) and used as an anchorage zone for dive- and small fishing boats. The features of the reef slope are comparable to those of the two sites on Curaçao [33]. *In situ* water sampling was conducted on SCUBA. The simple and inexpensive point sampler (SIP) system [34], the so-called VacuSIP system designed by G. Yahel was slightly modified (see Fig. 1; for detailed description see http://web.uvic.ca/~yahel/GYWS/Other/VacuSIP%20usage%20and%20makeup.pdf) and used for the *in situ* measurement of the difference in DOC concentration and bacterial abundance between a pair of inhaled and exhaled water samples mediated by a sponge. This difference provided a measure of the net retention (or production) of a waterborne compound by the animal [35]. The sampling system used here consisted of two separate VacuSIP samplers attached to a stand which allowed simultaneous sampling of water inhaled and exhaled by the sponge (Fig. 1). Each sampler consisted of PEEK (polyetheretherketone) tubing (1/16”×25 μm, UpChurch Scientific) with a syringe needle connected to a male luer connector (IDEX Health and Science, P-655 1/4-28) at its distal end (outlet). Samplers were attached to a flexible arm so that the proximal end (inlet) of one sampler could be positioned in the osculum (excurrent aperture; Ex) and another one (In) outside of the osculum at a distance of approximately 20 cm from the inhalant surface (to ensure sampling of ambient water without contamination from substances emitted from the surface of the sponge). After positioning the VacuSIP, it was left untouched for at least 3 min to minimize possible disturbance effects that could have occurred during the installation of the device. Evacuated vials (Vacuette, 9 mL, no additive, Greiner Bio-One GmbH) were used to collect bacterial abundance samples and pre-combusted (4 h at 450°C) Epa vials (40 mL) were used to collect samples for DOC concentration. Vials were connected to the samplers by piercing their septa with the syringe needle. The pressure difference between the external water and the neutral (Epa vials) or evacuated (vacuettes) vials ensured that water flowed into the container during sampling. For DOC sampling, an inline stainless steel filter holder (13 mm, Swinney, Pall) with a pre-combusted (4 h at 450°C) GF/F filter (Whatmann, 0.7 μm) was added.

To avoid contamination of the sampled water with ambient water, the VacuSIP water sampling rate was kept lower than the pumping – excurrent jet – rate of the sponge [35]. Therefore, the excurrent jet rate of each sponge was determined prior to sampling using the dye-front speed (DFS) technique [29]. A cut-open 15 mL Falcon tube (length 95 mm; diameter: 14 mm) was aligned with the osculum (diameter: 4–15 mm, Table 1) of the sponge (without touching it). A dye was released between osculum and tube and its movement with the excurrent jet through the tube was video-taped (three to five times). The resulting water transport speed (cm s^−1^) was multiplied with the cross-section area of tube (cm^2^) to yield the excurrent jet rate (mL min^−1^). During the In and Ex sampling the time to fill the containers was recorded to calculate the rate at which water was sampled. Mean sampling rate (±SD) for *Siphonodictyon* sp. and *C. delitrix* was 2.9±1.2 and 1.9±0.3 mL min^−1^ (ESM Table S1), respectively, which was two orders of magnitude less than the sponges' excurrent jet rate (table 1).

**Table 1:** Oscule diameter, water transport speed and excurrent jet rate for *Siphonodictyon* sp. and *Cliona delitrix*.

Species	ID	Oscule diameter (cm)	Water transport speed (cm s^−1^)	Excurrent jet rate (mL min^−1^)
*Siphonodictyon* sp.	S1	0.7	5.0	466.1
	S2	0.7	2.9	263.8
	S3	0.9	6.9	637.3
	S4	1.1	7.6	706.3
	S5	0.7	4.7	431.0
	S6	0.5	4.7	431.0
	S7	0.4	4.6	420.3
	S8	0.5	7.9	727.4
**Average (±SD)**		**0.7±0.2**	**5.5±1.8**	**510.4±162.6**
*Cliona delitrix*	C1	1.5	4.4	404.1
	C2	1.4	6.3	577.3
	C3	1.4	4.6	427.9
	C4	1.2	4.7	431.0
	C5	1.0	8.8	808.2
	C6	1.4	3.9	359.2
	C7	1.0	3.9	363.7
	C8	1.0	4.0	371.8
	C9	1.5	4.8	440.8
	C10	1.1	4.4	404.1
**Average (±SD)**		**1.1±0.2**	**5.0±1.5**	**458.8±137.7**

Prior to sampling, VacuSIP samplers were cleaned by flushing the sampler consecutively with 30 mL HCl (5%; except stainless steel filter holders to avoid corrosion), 30 mL MQ, and 30 mL Decon 90 (Decon Laboratories Limited; 5%). After *in situ* VacuSIP installment system samplers were flushed with 30 mL ambient seawater prior to sampling.

### Processing of samples

Water samples were processed within 1h after sampling. Samples for DOC concentration (20 mL) were acidified with 6–7 drops of concentrated HCl (38%) to remove inorganic carbon and stored in the dark at 4°C until analysis. DOC concentrations were measured using the high-temperature catalytic oxidation (HTCO) technique in a total organic C analyzer (TOC-VCPN; Shimadzu). The instrument was calibrated with a standard addition curve of Potassium Phthalate (0; 25; 50; 100; 200 µmol C L^−1^). Consensus Reference Materials (CRM) provided by Hansell and Chen of the University of Miami (Batch 12; 2012; 41–44 µmol C L^−1^) were used as positive controls for our measurements. Concentrations measured for the batch gave average values (±SD) of 45±2 µmol C L^−1^. Average analytical variation of the instrument was <3% (5–7 injections per sample).

Samples for bacterial abundance (9 mL) were fixed in 4% paraformaldehyde (PFA) and filtered over a 0.2 μm polycarbonate filter (Millipore, 25 mm), supported by a 0.45 μm HA filter (Millipore, 25 mm). The filters were air-dried and stored in Eppendorf tubes at −20°C. Prior to bacterial cell counts, filters were mounted on a microscopy slide in a DAPI-mix. Bacterial numbers were counted using an epifluorescence microscope (Zeiss Axioplan; 1000×). Per slide 10 grids (36×36 μm, divided into 10 rows and columns) were counted or up to a minimum of 200 bacteria.

### Data analysis

Differences in DOC concentration and bacterial abundance between In and Ex water samples were tested using the Wilcoxon Signed Rank test. To convert bacterial numbers to a corresponding amount of carbon biomass, a conversion factor for coastal bacteria of 30 fg per bacterial cell was used [36]. Net uptake (or release) rates are traditionally reported per unit of animal mass or volume. Yet, coral-excavating sponges such as *Siphonodictyon* sp. and *C. delitrix* live inside the substrate, which makes the quantification of such units difficult. Therefore, we followed the recommendation of Yahel et al. [35] and standardized fluxes to the excurrent jet rate. Uptake rates were calculated as the difference in concentration of an In-Ex pair (Δ concentration_In-Ex_) multiplied with the respective excurrent jet rate:



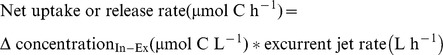
The TOC pool is comprised of DOC and particulate organic carbon (POC). In tropical reef waters POC consists mainly of phytoplankton and bacterioplankton. However, phytoplankton concentrations were not directly measured. Generally, the contribution of phytoplankton carbon to the total carbon pool in tropical waters is low and roughly equal [37]–[39] or lower than bacterioplankton carbon (BC) [40], [41]. To quantify the contribution of DOC and POC to TOC we followed the formula suggested by de Goeij et al. [30]:
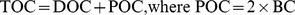



## Results

### Ambient DOC concentrations and net sponge DOC removal

**Figure 2:**
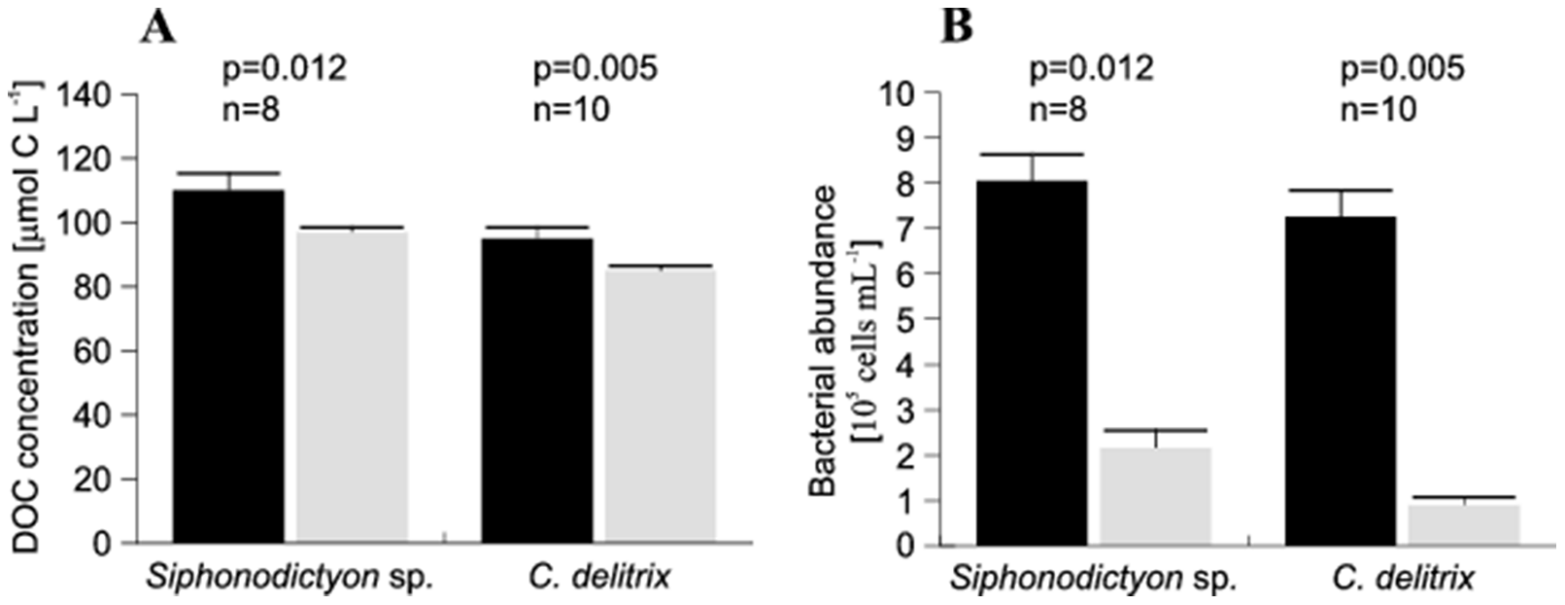
Average DOC (A) and bacterial abundance (B) in the inhaled (black) and exhaled (grey) water of *Siphonodictyon* sp. and *C. delitrix*. Error bars indicate SE. P values (Wilcoxon Signed Rank) indicate significance level of the difference in the concentrations between the inhaled and exhaled samples in n pairs of InEx samples.

**Figure 3:**
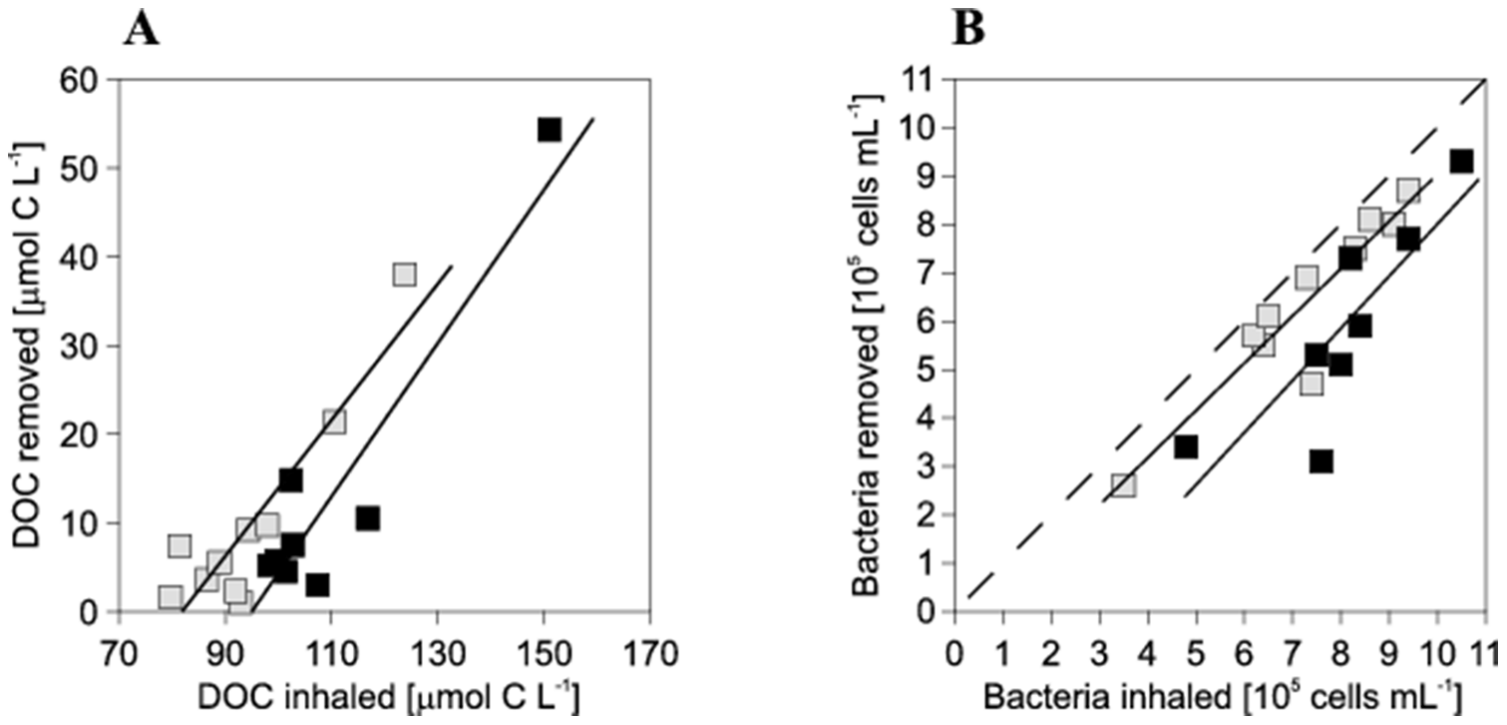
Removal of DOC (A) and bacterial cells (B) by *Siphonodictyon* sp. (black) and *C. delitrix* (grey) plotted against ambient (inhaled) concentrations. Both species responded linearly to elevated DOC (R^2^ = 0.88; p = 0.004 and R^2^ = 0.84; p = 0.002) and bacterial concentrations (R^2^ = 0.72; p = 0.045 and R^2^ = 0.87; p = 0.001) within the full concentration range encountered. Dashed line represents 100% bacterial removal.

Ambient DOC concentrations (mean±SD, derived from inhaled water) on Curaçao were 110±18 μmol L^−1^ and 95±5 μmol L^−1^ on Bonaire. POC concentrations were 4±1 μmol L^−1^ on both islands, so that ambient TOC concentrations were 114±18 and 99±13 μmol C L^−1^, for Curaçao and Bonaire, respectively. Both sponge species significantly removed amounts of DOC from the seawater pumped through their aquiferous system (Fig. 2A). DOC concentrations in the exhalant water were reduced by 13±17 μmol C L^−1^ for *Siphonodictyon* sp. (Wilcoxon Signed Rank: Z = −2.521, *n* = 8, *p* = 0.012) and 10±12 μmol C L^−1^ for *C. delitrix* (Wilcoxon Signed Rank: Z = −2.803, *n* = 10, *p* = 0.005), respectively, compared to the inhalant water. The majority of the TOC removed by the two coral-excavating sponges –82% (*Siphonodictyon* sp.) and 76% (*C. delitrix*) – consisted of DOC. The amount of DOC removed by both coral-excavating sponge species increased linearly with increasing ambient DOC concentrations (*Siphonodictyon* sp.: R^2^ = 0.88, *p* = 0.004; *C. delitrix*: R^2^ = 0.84, *p* = 0.002) encountered during the experiments (*Siphonodictyon* sp.: 98–151 μmol C L^−1^; *C. delitrix*: 80–124 μmol C L^−1^) (Fig. 3A). This indicates that no threshold or saturation effect occurred for the aforementioned ranges of ambient DOC concentrations.

### Ambient bacterial abundance and net sponge bacterial removal

Ambient bacterial abundance (mean±SD, derived from inhaled water) on Curaçao (8.0±1.6×10^5^ cells mL^−1^) and Bonaire (7.3±1.8×10^5^ cells mL^−1^) corresponded to 2.0±0.4 and 1.8±0.4 μmol C L^−1^, respectively. Both, *Siphonodictyon* sp. and *C. delitrix* significantly reduced ambient bacterial concentrations by 5.89±2.11×10^5^ (Wilcoxon Signed Rank: Z = −2.521, n = 8, p = 0.012) and 6.36±1.84×10^5^ cells mL^−1^ (Wilcoxon Signed Rank: Z = −2.803, n = 10, p = 0.005), respectively (Fig. 2B). Bacteria removal efficiency was 72±15% and 87±10%, but despite these high efficiencies, bacterial removal accounted for only 9% (*Siphonodictyon* sp.) and 12% (*C. delitrix*) of the total TOC removal. Similar to the uptake of DOC, the number of bacteria cells removed by excavating sponges from the surrounding water increased linearly with increased cell abundance in the water column (*Siphonodictyon* sp.: R^2^ = 0.72, p = 0.0045; *C. delitrix*: R^2^ = 0.87, p = 0.001; Fig. 3B). Across the range of ambient bacterial concentrations encountered (*Siphonodictyon* sp.: 4.8–10.5×10^5^ cells mL^−1^; *C. delitrix*: 3.5–9.4×10^5^ cells mL^−1^) no indication of a threshold or saturation concentration occurred.

### Sponge DOC and bacterial uptake rates

Water transport speed of *Siphonodictyon* sp. and *C. delitrix* were comparable at 5.4±1.8 and 5.0±1.5 cm s^−1^, respectively (table 1). And despite of 1.8 times larger mean oscule diameter for *C. delitrix* (table 1), mean excurrent jet rates were comparable as well (*Siphonodictyon* sp.: 510.4.5±162.6 mL min^−1^; *C. delitrix*: 458.8±137.7 mL min^−1^). Mean DOC uptake rate of *Siphonodictyon* sp. was 461±773 μmol C h^−1^ and, therefore, 1.3 times higher than that of *C. delitrix* (354±562 μmol C h^−1^) (table 2).

**Table 2:** Mean DOC and bacteria uptake rates (±SD) of *Siphonodictyon* sp. and *Cliona delitrix* standardized to excurrent jet rate.

species	DOC (μmol C h^−1^)	Bacteria (10^10^ cells h^−1^)	Bacteria (μmol C h^−1^)
*Siphonodictyon* sp.	461±773	1.8±0.9	46.0±21.2
*C. delitrix*	354±562	1.7±0.6	42.5±14.0

Mean bacteria uptake rate of *Siphonodictyon* sp. and *C. delitrix* were 1.8±0.9×10^10^ and 1.7±0.6×10^10^ cells h^−1^, respectively (table 2). These bacterial removal rates correspond to a BC uptake of 46.0±21.2 and 42.5±14.0 μmol C h^−1^. Therefore, DOC represents 83 and 81% of the TOC taken up by *Siphonodictyon* sp. and *C. delitrix* per hour.

## Discussion

The coral-excavating sponges *Siphonodictyon* sp. and *C. delitrix* are both lacking photosynthetic symbionts ([42], pers. comm. C.H.L. Schönberg) and can therefore be considered as classic heterotrophs that depend on the uptake of organic matter as carbon and energy source. This study demonstrates that both species mainly rely on DOC uptake to meet their carbon demand. Despite high bacterial retention efficiencies, these sponges can be typified as DOM-feeders, retaining 83% and 81% of the TOC taken up in the form of DOC. This contribution of DOC in their daily diet is in the same range, and only slightly lower, than that reported for non-excavating sponges, such as the reef sponges *Theonella swinhoei* (Gray, 1868), *Halisarca caerulea* (Vacelet and Donadey, 1987), *Mycale microsigmatosa* (Arndt, 1927) and *Merlia normani* (Kirkpatrick, 1908) [29], [30]. Our results further suggest that, similar to bacteria (Fig. 3B) or phytoplankton (e.g., [29], [34]), sponges can efficiently take up DOC across a wide range of ambient DOC concentrations (Fig. 3A). This indicates that these sponges are well adapted to utilize DOC as food source [34], [43]. DOC uptake by sponges has been confirmed in an increasing number of species belonging to various orders of Demospongiae [29]–[32] and one order of Hexactinellida [44] (ESM table S2).

### Sponge DOC and bacterial uptake rates

Since uptake rates were standardized to the excurrent jet rate and not to biomass or volume, results are primarily discussed in comparison to *T. swinhoei* in Yahel et al. [29], where necessary parameters are available. Largely similar retention efficiencies (DOC: 11–12%; bacteria: 72–87%) and water transport speeds (table 1) resulted in comparable DOC and bacterial uptake rates in *Siphonodictyon* sp. and *C. delitrix* (table 2). Yet, the DOC uptake rates were approximately three times higher than reported for *T. swinhoei* (DOC: 138 μmol C h^−1^). Similarly, bacterial uptake rates were twice as high for the excavating sponge species as for *T. swinhoei* (bacteria: 1.0×10^10^ cells h^−1^). This difference in uptake rates can be explained by a lower volume of water passing through *T. swinhoei* as indicated by a 2 times lower excurrent jet rate (230 mL min^−1^). Environmental factors (e.g. sediment in the water column) and mechanical stimuli are reported to reduce and/or arrest the pumping activity of sponges [45], [46]. Since the excurrent jet rate was only measured prior to the sampling it cannot be excluded that it varied during the sampling, which could explain the overall high variability in bacterial and DOC uptake rates in both excavating sponge species tested. Assuming an average daily pumping activity of 12 h [24] yields a TOC uptake of 6.6 and 5.2 mmol C d^−1^ for *Siphonodictyon* sp. and *C. delitrix*, respectively. It should be noted that the here presented uptake rates are given per excurrent jet and that both species are multi-oscular sponges and have therefore multiple excurrent jets. *C. delitrix* can grow up to a size of 1 m across with >30 oscules per specimen (B. Mueller pers. obs.). At Playa Lechi, our study site on Bonaire, the abundance of *C. delitrix* was with 0.03 individuals m^−2^ relatively low (Y. Mulders pers. obs.). However, densities of up to 0.23 and 0.54 individuals m^−2^ were reported for Grand Cayman and San Andrés, Columbia, respectively [10], [47]. When occurring in such high densities, *C. delitrix* is likely to have a significant effect on benthic carbon cycling by ingesting POC and especially DOC from the ambient water. Abundance data for *Siphonodictyon* sp. are rare, but with approximately 0.23 individuals m^−2^ on the south-western coast of Curaçao this species is quite common (B. Mueller and F.C. Van Duyl pers. obs.). However, specimens are comparable small at 48 cm^2^. *Siphonodictyon coralliphagum* (Rützler, 1971) is reported to grow up to a size of 600 cm^2^ [48] and individuals of >0.5 m across can be regularly encountered on Cozumel, Mexican Caribbean (B. Mueller pers. obs.). Therefore, also *Siphonodictyon* sp. might have a significant effect on benthic carbon cycling, when occurring in high densities and large sizes.

### Potential effect of a coral-algal phase shift on coral-excavating sponges

The ability of sponges to take up and assimilate DOC [32], [49] has been proposed to be crucial to maintain biodiversity and high productivity on tropical coral reefs [32]. In the so-called “sponge loop”, analogously to the microbial loop, sponges make energy and nutrients stored in the dissolved organic matter (DOM) pool available to the benthic food web via DOM assimilation and subsequent detritus production by the sponges. Our study now shows that excavating sponges most likely also participate in the sponge loop, although it remains unclear to what extent these sponges produce detritus and what the nutritional value of this detritus is to other reef fauna. However, it is very clear that there is a current increase in the abundance of coral-excavating sponges throughout the Caribbean (e.g., [9], [42]). This increase is commonly attributed to a combination of an increase in the availability of new substrate due to coral declines [9], [50] and an increase in food availability as a result of eutrophication and pollution [8]–[10]. Regarding the latter, being suspension feeders, coral-excavating sponges were considered to benefit from elevated concentration of particulate resources, such as phytoplankton and bacteria (e.g., [8], [9], [47]). However, here we could show that coral-excavating sponges mainly rely on DOC to meet their carbon demand. Thus, an increase in DOC production, or quality, on coral reefs is likely to be beneficial for them. Shifts in the benthic reef community have caused major changes in the production and cycling of organic matter on reefs [51], [52]. Due to anthropogenic disturbances benthic algae are increasing at the expense of scleractinian corals on most coral reefs throughout the Caribbean region (e.g., [53]–[55]). Both, scleractinian corals and benthic algae release a substantial amount of their photosynthetically fixed carbon as organic matter in the surrounding water [56]–[58]. However, benthic algae are reported to release more DOM than corals (e.g., [52], [56], [59]) and algal-derived DOM appears to be of a higher quality [52], [60]. Sponges, including excavating species, could therefore benefit in two ways from an increase in DOM production and quality due to the shift in benthic communities: (1) directly via uptake of DOM and (2) indirectly by feeding on the heterotrophic planktonic microbial community, which is fueled by the DOM release of benthic algae. However, the competition between algae and (coral-excavating) sponges is controversial. A general negative correlation between the abundance of benthic algae and phototrophic excavating sponges was observed in the Mediterranean and on the Great Barrier Reef [61], [62]. Furthermore, competition for space between benthic algae and the phototrophic coral-excavating sponge *Cliona tenuis* (Zea and Weil, 2003) has been reported in the Caribbean [2], [63]. Despite possible DOM consumption by this sponge, the beneficial effects of the availability of algal-DOM might be reduced or even eliminated by the effects of sunlight shading by benthic algae, reducing the photosynthetic performance of the sponge [61], [62]. Interestingly, *C. tenuis* was reported to advance over turf algae [2], [64], which are known to release high amounts of DOC (e.g., [57], [59]) and do not shade the sponge. Moreover, a coexistence of sponges and benthic algae jointly dominating the benthic community was found on several Caribbean reefs and is suggested to become more frequent with increasing reef degradation [65], [66].

Here we could show that the coral-excavating sponges *Siphonodictyon* sp. and *C. delitrix* are capable of consuming DOC and mainly rely on DOC to meet their organic carbon demand. This suggests that coral-excavating sponges are likely to benefit from an increase in DOC production and quality as a result of the ongoing coral-algal phase shift.

## Supporting Information

Table S1 Sampling dates, locations, depth as well as sampling rates for *Siphonodictyon* sp. and *Cliona delitrix*.
(PDF)

Table S2 Confirmed DOC feeding by sponges.
(PDF)
