# Supplementary material for: Natural Diet of Coral-Excavating Sponges Consists Mainly of Dissolved Organic Carbon (DOC)
Source: PLoS One. 2014 Feb 25;9(2):e90152. doi: 10.1371/journal.pone.0090152 (PMC3934968; doi:10.1371/journal.pone.0090152)
Supplement: Table S1 — Sampling dates, locations, depth as well as sampling rates for Siphonodictyon sp. and Cliona delitrix. (PDF) [file pone.0090152.s001.pdf]

Table S1. Sampling dates, locations, depth as well as sampling rates for *Siphonodictyon* sp. and *Cliona delitrix*.

Species	ID	Date	Location	Depth (m)	Sampling rate (ml min^{-1})
Siphonodictyon sp.	S1	09/05/2013	Daaibooi/Curaçao	19	1.8
	S2	09/05/2013	Playa Jeremy/Curaçao	17	1.3
	S3	10/05/2013	Playa Jeremy/Curaçao	20	4.5
	S4	12/05/2013	Playa Jeremy/Curaçao	20	1.7
	S5	12/05/2013	Playa Jeremy/Curaçao	18	2.8
	S6	27/05/2013	Daaibooi/Curaçao	19	3.9
	S7	27/05/2013	Daaibooi/Curaçao	18	2.9
	S8	27/05/2013	Daaibooi/Curaçao	18	2.5
**Average (±SD)**				**19 ± 1**	**2.9 ± 1.2**
Cliona delitrix	C1	20/05/2013	Playa Lechi/Bonaire	12	2.0
	C2	20/05/2013	Playa Lechi/Bonaire	15	2.7
	C3	20/05/2013	Playa Lechi/Bonaire	14	1.8
	C4	20/05/2013	Playa Lechi/Bonaire	14	1.8
	C5	20/05/2013	Playa Lechi/Bonaire	13	1.7
	C6	20/05/2013	Playa Lechi/Bonaire	16	1.6
	C7	21/05/2013	Playa Lechi/Bonaire	14	1.6
	C8	21/05/2013	Playa Lechi/Bonaire	14	2.1
	C9	21/05/2013	Playa Lechi/Bonaire	12	1.6
	C10	21/05/2013	Playa Lechi/Bonaire	12	1.7
**Average (±SD)**				**13 ± 1**	**1.9 ± 0.3**
