# Supplementary material for: Natural Diet of Coral-Excavating Sponges Consists Mainly of Dissolved Organic Carbon (DOC)
Source: PLoS One. 2014 Feb 25;9(2):e90152. doi: 10.1371/journal.pone.0090152 (PMC3934968; doi:10.1371/journal.pone.0090152)
Supplement: Table S2 — Confirmed DOC feeding by sponges. (PDF) [file pone.0090152.s002.pdf]

Table S2. Confirmed DOC feeding by sponges.

**class**	**order**	**species**	**environment**	**Reference**
Demospongia	Agelasida	*Agelas oroides*	Mediterranean	Ribes et al. 2012
	Chondrosida	*Chondrosia reniformis*	Mediterranean	Ribes et al. 2012
		Chondrilla caribensis	Caribbean; coral reef	de Goeij et al. 2013
		Halisarca caerulea	Caribbean; coral reef	de Goeij et al. 2008
	Hadromerida	*Cliona delitrix*	Caribbean; coral reef	this study
	Halichondrida	*Higginsia thielei*	NE Atlantic; deep water coral reef	van Duyl et al 2008
		Scopalina ruetzleri	Caribbean; coral reef	de Goeij et al. 2013
	Haplosclerida	*Haliclona implexiformis*	Caribbean; coral reef	de Goeij et al. 2013
		Siphonodictyon sp.	Caribbean; coral reef	this study
	Lithistida	*Theonella swinhoei*	Red Sea; coral reef	Yahel et al. 2003
	Poecilosclerida	*Merlia normani*	Caribbean; coral reef	de Goeij et al. 2008
		Mycale microsigmatosa	Caribbean; coral reef	de Goeij et al. 2008
Hexactinellida	Lyssacinosida	*Nodastrella nodastrella*	NE Atlantic; deep water coral reef	van Duyl et al 2008

References:

De Goeij JM, Van den Berg H, Van Oostveen MM, Epping EHG, Van Van Duyl FC (2008) Major bulk dissolved organic carbon (DOC) removal by encrusting coral reef cavity sponges. Mar Ecol Prog Ser 357: 139-151

De Goeij JM, Van Oevelen D, Vermeij JMA, Osinga R, Middelburg JJ, De Goeij AFPM, Admiraal W (2013) Surviving in a marine desert: the sponge loop retains resources within coral reefs. Science 342: 108-110

Ribes M, Jimenez E, Yahel G, Lopez-Sendino P, Diez B, Massana R, Sharp JH, Coma R (2012) Functional convergence of microbes associated with temperate marine sponges. Environ Microbiol 14: 1224-1239

Van Duyl FC, Hegeman J, Hoogstraten A, Maier C (2008) Dissolved carbon fixation by sponge-microbe consortia of deep water coral mounds in the northeastern Atlantic Ocean. Mar Ecol Prog Ser 358: 137-150

Yahel G, Sharp JH, Marie D, Haese C, Genin A (2003) *In situ* feeding and element removal in the symbiont-bearing sponge *Theonella swinhoei*: bulk DOC is the major source for carbon. Limnol Oceanogr 48:141–149
